# Diagnositic value of pelvic enthesitis on MRI of the sacroiliac joints in enthesitis related arthritis

**DOI:** 10.1186/s12969-015-0045-5

**Published:** 2015-11-10

**Authors:** N. Herregods, J. Dehoorne, E. Pattyn, J. L. Jaremko, X. Baraliakos, D. Elewaut, J Van Vlaenderen, F. Van den Bosch, R. Joos, K. Verstraete, L. Jans

**Affiliations:** Department of Radiology and Medical Imaging, Ghent University Hospital, De Pintelaan 185, 9000 Gent, Belgium; Department of Pediatric Rheumatology, Ghent University Hospital, De Pintelaan 185, 9000 Gent, Belgium; Department of Radiology & Diagnostic Imaging, University of Alberta Hospital, 8440-112 Street, Edmonton, T6G 2B7 Alberta Canada; Rheumazentrum Ruhrgebiet, Ruhr-University Bochu, Claudiusstr. 45, 44649 Herne, Germany; Department of Rheumatology, Ghent University Hospital, De Pintelaan 185, 9000 Gent, Belgium

**Keywords:** MRI, Enthesitis-related arthritis, Sacroiliitis, Enthesitis, Pelvic

## Abstract

**Background:**

To determine the prevalence and diagnostic value of pelvic enthesitis on MRI of the sacroiliac (SI) joints in enthesitis related arthritis (ERA).

**Methods:**

We retrospectively studied 143 patients aged 6–18 years old who underwent MRI of the SI joints for clinically suspected sacroiliitis between 2006–2014. Patients were diagnosed with ERA according to the International League of Associations for Rheumatology (ILAR) criteria. All MRI studies were reassessed for the presence of pelvic enthesitis, which was correlated to the presence of sacroiliitis on MRI and to the final clinical diagnosis. The added value for detection of pelvic enthesitis and fulfilment of criteria for the diagnosis of ERA was studied.

**Results:**

Pelvic enthesitis was seen in 23 of 143 (16 %) patients. The most commonly affected sites were the entheses around the hip (35 % of affected entheses) and the retroarticular interosseous ligaments (32 % of affected entheses). MRI showed pelvic enthesitis in 21 % of patients with ERA and in 13 % of patients without ERA. Pelvic enthesitis was seen on MRI in 7/51 (14 %) patients with clinically evident enthesitis, and 16/92 (17 %) patients without clinically evident enthesitis. In 7 of 11 ERA-negative patients without clinical enthesitis but with pelvic enthesitis on MRI, the ILAR criteria could have been fulfilled, if pelvic enthesitis on MRI was included in the criteria.

There is a high correlation between pelvic enthesitis and sacroiliitis, with sacroiliitis present in 17/23 (74 %) patients with pelvic enthesitis.

**Conclusions:**

Pelvic enthesitis may be present in children with or without clinically evident peripheral enthesitis. There is a high correlation between pelvic enthesitis and sacroiliitis on MRI of the sacroiliac joints in children. As pelvic enthesitis indicates active inflammation, it may play a role in assessment of the inflammatory status. Therefore, it should be carefully sought and noted by radiologists examining MRI of the sacroiliac joints in children.

## Background

Spondyloarthropathies (SpA) are a group of related inflammatory diseases characterized by enthesitis and arthritis. There is a strong association with the human leukocyte antigen (HLA) B27 [1–3]. The presentation of juvenile spondyloarthropathies (JSpA) differs in children and adults; most notably, spinal involvement is uncommon, while hip arthritis and enthesitis are frequently seen in juvenile-onset disease, with the calcaneal insertion, the Achilles tendon and plantar fascia, being the most commonly affected sites [4–8].

Currently, the classification of SpA in adults and children is different; using the International League of Associations for Rheumatology (ILAR) system for juvenile idiopathic arthritis, most childhood SpA is classified as Enthesitis-Related Arthritis (ERA) [6, 7].

Entheses are sites where tendons, ligaments, capsules or fascia are attached to bone providing a mechanism for reducing stress at the bony interface. According to the concept of ‘enthesis organ’ and ‘functional entheses’ , inflammatory changes in the immediate vicinity of the insertion are also considered to represent enthesitis [9–11].

Enthesitis is a primary feature of JSpA but is often difficult to diagnose clinically [12]. Deep-seated entheses are difficult to palpate. MRI is excellent for demonstrating enthesitis, depicting not only bone marrow oedema but also soft tissue inflammation and joint effusion/bursitis [10], as has been shown in adult SpA patients [10, 13, 14]. In patients with enthesitis-related enthesitis (ERA), it has been shown that the number of active entheses and joints at onset can predict sacroiliitis at follow-up [15]. Assessment of entheses on pelvic MRI therefore can be of great value.

Routine MRI detects active and structural lesions of sacroiliitis via semicoronal T1 and short-tau inversion recovery (STIR) sequences. Some pelvic entheses can also be evaluated on these sequences and on the axial sequences frequently obtained [10, 13].

As in adult SpA, early diagnosis in children has become more important to alter the course of the disease [16, 17]. This requires early detection of inflammatory changes. Unlike adults, there is little published regarding MRI assessment of sacroiliitis and pelvic findings such as enthesitis in children.

The aim of this study is to determine the prevalence and diagnostic value of pelvic enthesitis on MRI of the SI joints in enthesitis-related arthritis.

## Methods

### Study group

A retrospective study of all pediatric MRI of the sacroiliac joint from January 2006 to October 2014 was approved by the institutional ethics committee. All patients were sent from the pediatric rheumatology department in a tertiary care center and were referred for MRI of the SI joints with sacroiliac joint tenderness or clinical (inflammatory) low back pain (IBP) suspected for sacroiliitis, according to an expert opinion of the pediatric rheumatologists. We defined IBP as history of back pain for at least three months with either (not and): insidious onset or improvement with exercise or no improvement with rest or pain at night (with improvement upon getting up). Patients were only included when age at onset of the disease was < 16 years.

We recorded from the clinical files if patients fulfilled the ILAR (International League of Associations for Rheumatology) classification criteria for enthesitis-related arthritis (ERA) [7]: Arthritis plus Enthesitis, OR, Arthritis or Enthesitis, plus at least two of the following: presence of a history of sacroiliac joint tenderness and/or inflammatory lumbosacral pain, presence of HLA-B27 antigen, onset of arthritis in a male over 6 years of age, acute (symptomatic) anterior uveitis, history of AS, ERA, sacroiliitis with IBD, reactive arthritis, or acute anterior uveitis in a first-degree relative. Exclusion criteria: Psoriasis or history of psoriasis in the patient or a first-degree relative; Presence of IgM RF on at least two occasions at least 3 months apart; Systemic JIA in the patient.

According to these criteria, two separate groups were composed, the first one with ERA-positive, the second one with ERA-negative children.

### Magnetic resonance imaging

MRI was performed on a body flexed array coil in a 1.5 T MRI unit (Avanto, Siemens Medical, Erlangen, Germany). Sequence protocol included: semicoronal (along long axis of the sacral bone perpendicular to the S2 vertebral body) T1-weighted turbo spin echo (TSE) (slice thickness (ST): 3 mm; repetition time/echo time (TR/TE): 368/20 ms); semicoronal short tau inversion recovery sequence (STIR) (ST: 3 mm; TR/TE/TI: 5030/67/150 ms); axial STIR (ST: 5 mm; TR/TE/TI: 7540/67/150 ms). As per ASAS guidelines, no contrast-enhanced pulse sequences were obtained [13].

### Image review

All MRI studies were reassessed. The MR images were reviewed for the presence of pelvic enthesitis by a pediatric radiologist with 11 years of experience (NH), and a musculoskeletal radiologist with 12 years of experience (LJ), who were blinded to clinical and other imaging findings.

**Fig. 1:**
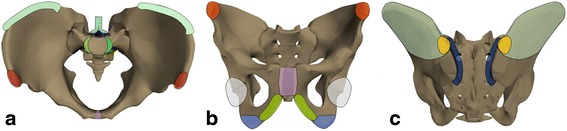
Sites of entheses of the pelvis. **a**. Longitudinal ligament insertion (*green*), vertebral posterior elements (*blue*), anterior superior iliac spine (*red*), symphysis pubis (*purple*), iliac crest (*cyan*). **b**. Anterior superior iliac spine (*red*), pubic symphysis (*purple*), ramus of pubis (*yellow*), ischial tuberosity (*blue*), hip (*light grey*). **c**. Retroarticular ligaments (*dark blue*), iliac crest and wing (*cyan*), posterior superior iliac spine (*orange*)

Entheses studied for presence of inflammation included (Fig. 1): a. the longitudinal ligaments (anterior and posterior) and ligaments of the posterior elements of visualised lumbar vertebra (supra- and interspinous), b. the interosseous ligaments in the retroarticular space of the SI joint (posterior of the synovial part of the joint), c. the entheses on the iliac crest and wing (attachment of gluteus maximus, medius and minimus, and transversus abdominis, quadratus lumborum and erector spinae muscles), d. the anterior (attachment of Sartorius and rectus femoris muscle) and e. posterior (attachment of piriform muscle) iliac spines, f. the pubic symphysis and g. the entheses of the hip. The entheses of the hip include the muscle attachments in the immediate vicinity, such as attachments of gluteus medius and minimus muscle to the greater and of the iliopsoas muscle to the lesser trochanter as well as the origin of the adductor muscles at the pubic and ischial bones [10].

Enthesitis was defined according to the MRI definition in the ASAS Handbook as hyperintense signal on T2 FS/STIR images at sites where ligaments and tendons attach to bone, including the retroarticular space (interosseous ligaments). The signal may extend to adjacent bone marrow and soft tissue [13]. Bone marrow edema (BME), peri-enthesal soft tissue inflammation and bursal fluid were evaluated [10, 13]. A binary score was used for each feature, i.e., 1 = present, 0 = absent.

**Fig. 2:**
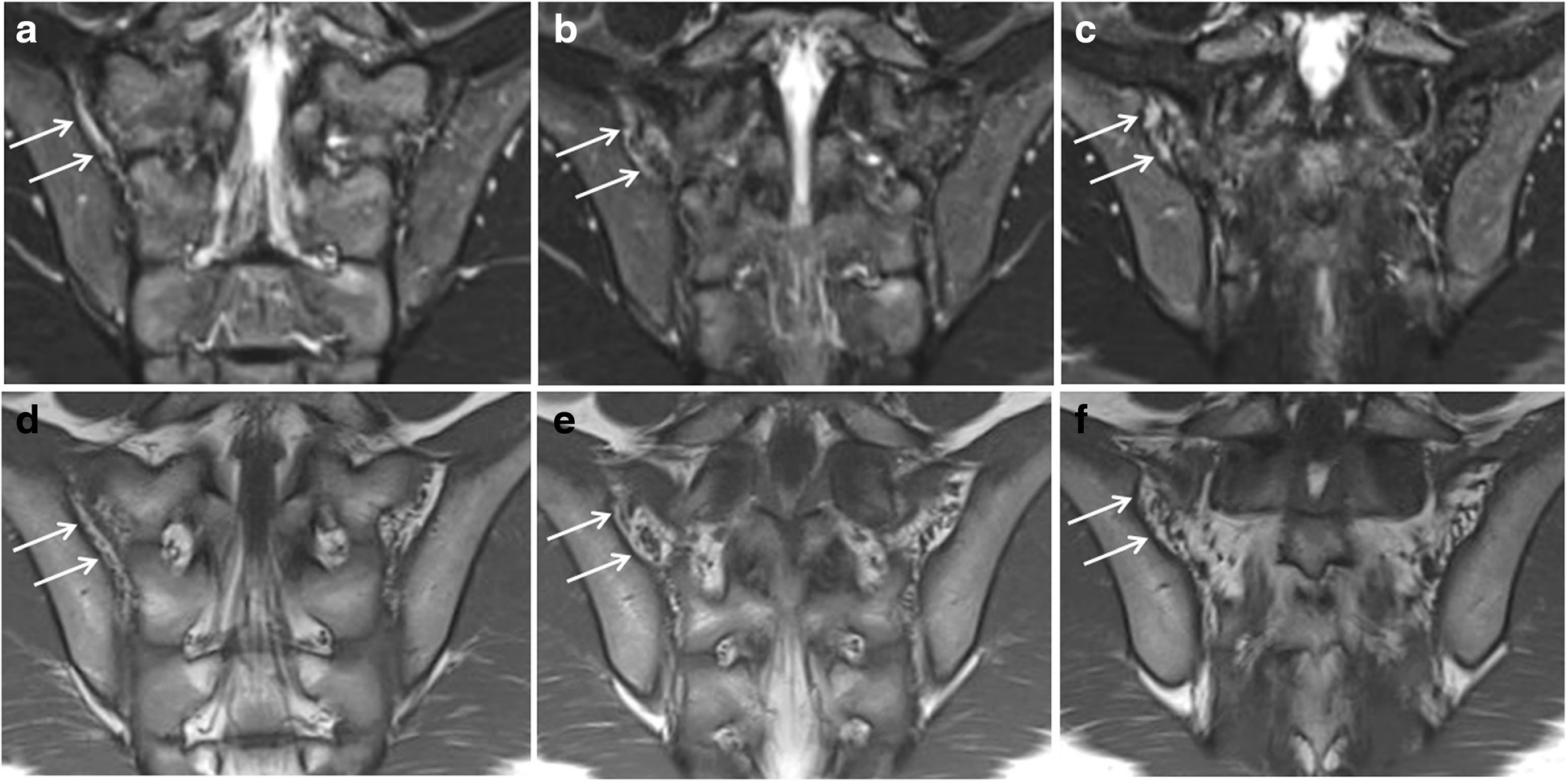
Enthesitis of the retroarticular interosseous ligaments in a 12-year-old boy with enthesitis-related arthritis. **a**-**c**. Consecutive semicoronal STIR MR images demonstrate enthesitis of the retroarticular interosseous ligaments on the right side (arrows). **d**-**f**. Corresponding semicoronal T1-weighted images demonstrate that the high signal is seen in the retroarticular fat tissue and not in the cartilaginous part of the SI joint

As for enthesitis in the retroarticular space of the SI joint, edema/high STIR signal can be seen in the ligamental/fibrous part of the SIjoint, which can be depicted on T1 images as the presence of multiple ligaments in the fatty matrix, as demonstrated in Fig. 2. BME of the SI joint in sacroiliitis is seen strictly in the synovial part of the joint as periarticular high signal.

**Fig. 3:**
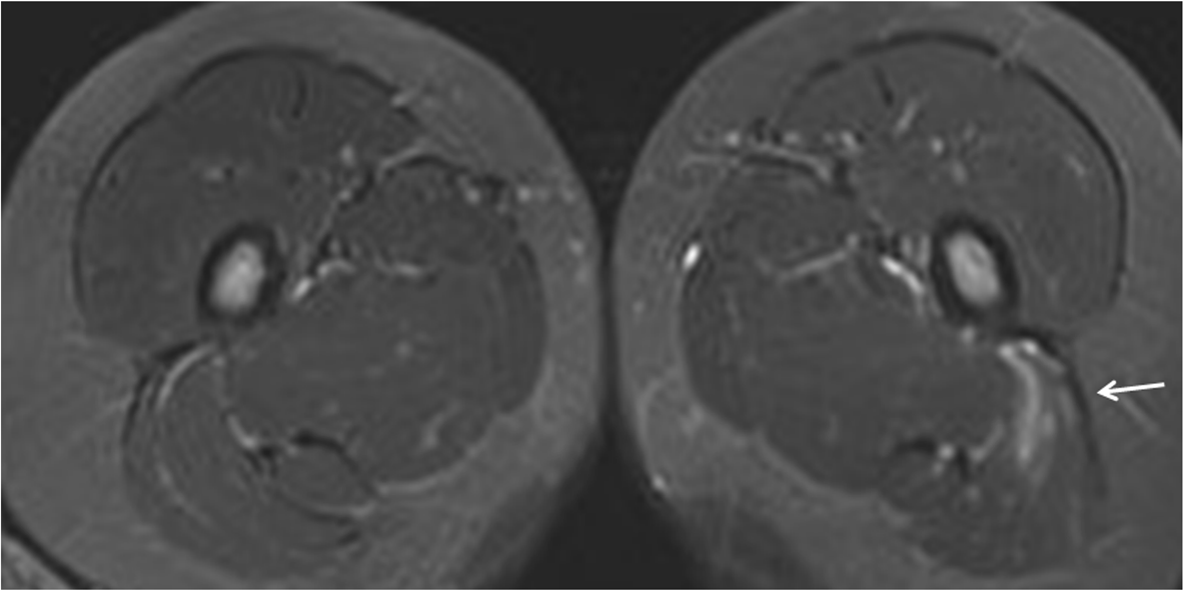
Enthesitis of the left gluteus maximus insertion in an 8-year-old girl with enthesitis-related arthritis on an axial STIR MR image (arrow)

**Fig. 4:**
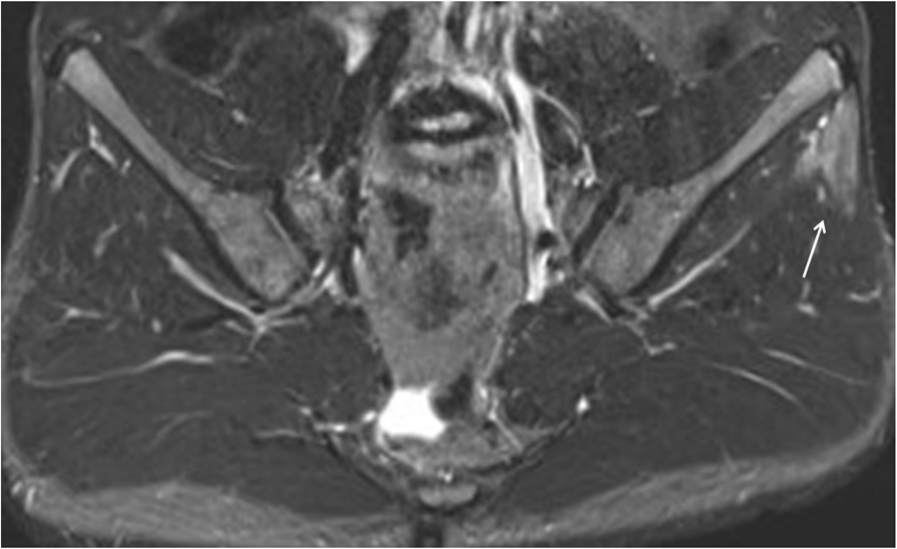
Enthesitis of the left gluteus medius origin in a 15-year-old boy with arthralgias (ERA-negative according to ILAR). Semicoronal STIR MR image of the SI-joint shows edema at the origin of the gluteus medius muscle at the iliac crest representing enthesitis

**Fig. 5:**
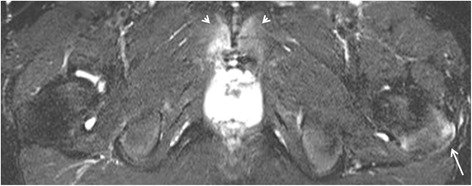
Enthesitis of the symphysis pubis and left greater trochanter in a 16-year-old boy with enthesitis-related arthritis. Axial STIR image shows high signal intensity in the marrow at the symphysis pubis (short arrows) representing osteitis, and at the left greater trochanter (arrow), representing enthesitis

**Fig. 6:**
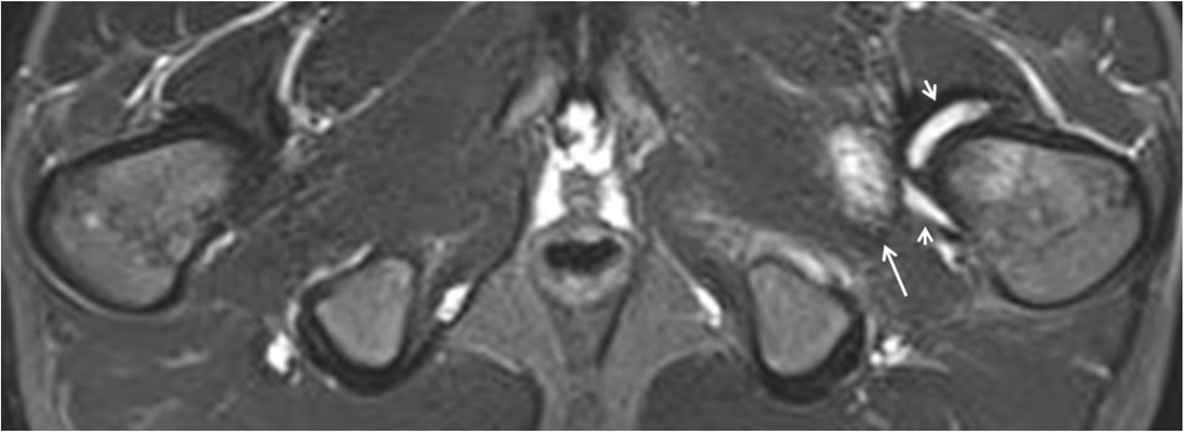
Enthesitis of the left external obturator muscle in a 14-year-old boy with enthesitis-related arthritis. Axial STIR image shows high signal intensity in the left external obturator muscle (arrow), representing hip enthesitis. Note also some fluid in the left hip joint (short arrows)

**Fig. 7:**
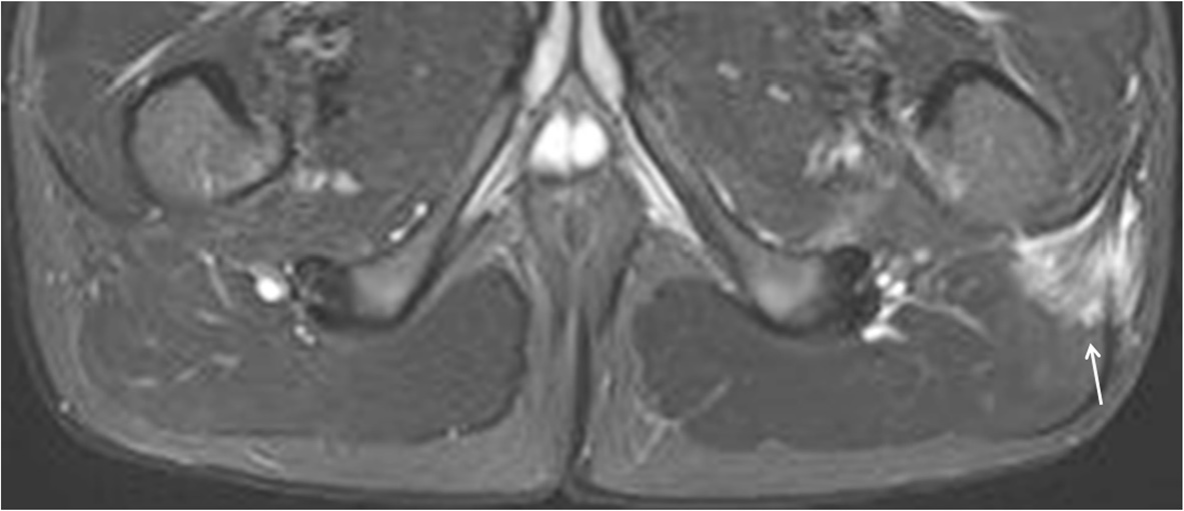
Enthesitis of the left gluteus maximus insertion in a 15-year-old boy with enthesitis-related arthritis. Axial STIR image demonstrates high signal in the left gluteus maximus insertion (arrow)

Representative images are presented in Figs. 2, 3,4, 5, 6 and 7.

### Statistical analysis

Statistical analysis was performed using software package SPSS 20.0 for Windows (SPSS, Chicago, IL, USA). Basic descriptive statistics were performed where appropriate: sensitivity and specificity of enthesitis for diagnosis of ERA were determined. Wilson score confidence intervals were calculated. Fisher Exact Test was used to examine the significance of the association between pelvic enthesitis and sacroiliitis on the one hand, and between pelvic enthesitis and the clinical diagnosis of ERA on the other hand.

With data obtained from independent readings of 40 randomly chosen MRI studies, κ values with standard errors were calculated in SPSS to assess agreement between the radiologists. κ values of 0–0.20 were considered to indicate slight agreement, values of 0.21–0.40 fair agreement, values of 0.41–0.60 moderate agreement, values of 0.61–0.80 substantial agreement, values of 0.81–0.99 almost perfect agreement, and a value of 1 perfect agreement [18].

## Results

### Study group

From January 2006 to October 2014, 143 patients with inflammatory low back pain and suspected sacroiliitis were sent for MRI of the sacroiliac joints. There were 52 (36 %) boys, 91 (64 %) girls, with a median age of 14.3 years (range 7–16).

Fifty-eight of 143 (40 %) patients were classified to have ERA based on ILAR criteria. In Table 1, clinical data were presented for all patients, and also separately for the two groups. Of all 143 patients, 38 % had arthritis, 34 % enthesitis (all peripheral, most frequently Achilles tendon), 46 % had sacroiliac joint tenderness, 43 % complained of inflammatory low back pain, and 34 % were HLA-B27 positive (23 % non determined).

**Table 1:** Summary of demographics, ILAR criteria and result of MRI of the sacroiliac joint

	TOTAL	ERA +	ERA
Female	91	30 (33 %)	61 (67 %)
Male	52	28 (54 %)	24 (46 %)
Arthritis	55	42 (76 %)	13 (24 %)
Enthesitis	48	43 (90 %)	5 (10 %)
SIjoint tenderness	66	33 (50 %)	33 (50 %)
Inflammatory LBP	62	36 (58 %)	26 (42 %)
HLA-B27	49 (33 ND)	30 (61 %) (3 ND)	19 (39 %) (30 ND)
Onset male >6	30	24 (80 %)	6 (20 %)
Acute anterior uveitis	7	3 (43 %)	4 (57 %)
History of AS, ERA, IBD related, ReA, AAU in first degree relative	23	11 (48 %)	12 (52 %)
Sacroiliitis on MRI	45	32 (71 %)	13 (29 %)
	143	58	85

(ERA = Enthesitis Related Arthritis, SI = sacroiliac, LBP = low back pain, HLA = Human Leukocyte Antigen, ND = Not determined, AS = Ankylosing Spondylitis, IBD = Inflammatory Bowel Disease, ReA = Reactive Arthritis, AAU = Acute Anterior Uveitis, MRI = Magnetic Resonance Immaging)

The diagnoses of the patients without ERA were summarized in Table 2. Five patients were considered positive for JSpA according to an expert opinion of our pediatric rheumatologists but did not fulfil the criteria. As the ILAR classification was considered the gold standard, they were classified as ERA-negative in our study. Of these 5 patients, 2 showed pelvic enthesitis as well as sacroiliitis on MRI.

**Table 2:** The diagnosis of the patients without ERA

Other diagnoses then ERA	
JIA oligoarticular	3
JIA polyarticular	3
Polyarthralgias mechanical	10
Auto-inflammatory syndrome	1
IBD related arthritis	2
Hyperlaxity syndrome	4
PsA	5
JSpA according to expert opinion	5
Undifferentiated/no clear diagnosis	52
	85 TOTAL

(JIA = Juvenile Idiopathic Arthritis, IBD = Inflammatory Bowel Disease, PsA = Psoriasis Arthritis, JSpA = Juvenile Spondylarthropathy)

One patient was diagnosed with lymphoma and was excluded from our study.

### Prevalence of enthesitis on MRI of the SI joints

Enthesitis was seen in 23 of 143 (16 %) patients. The prevalence of enthesitis of the different pelvic entheses is demonstrated in Table 3.

**Table 3:** Prevalence of hip involvement and enthesitis in different pelvic entheses

	N
Corner Inflammation	1
Posterior elements	2
Posterior iliac spine	3
Anterior iliac spine	1
Retroarticular ligaments	10
Iliac wing	1
Pubic symphysis	2
Hip entheses	11
TOTAL	31

In total, 31 entheses were affected in 23 patients (N = number of enthesitis)

The most commonly affected entheses were the entheses around the hip (35 % of affected entheses) and the retroarticular interosseous ligaments (32 % of affected entheses).

The κ values indicating interobserver agreement between the radiologists were very high (0.90), indicating very good agreement.

Of the 11 affected entheses of the hip, there was enthesitis at the greater trochanter insertion in 8, enthesitis at the external obturator insertion in 2, and enthesitis at the lesser trochanter insertion in 1. Two patients showed enthesitis at the greater trochanter concomitant with the posterior iliac spine, two patients showed enthesitis at the greater trochanter concomitant with the retroarticular interosseous ligaments, and one patient showed enthesitis at the lesser trochanter insertion concomitant with the external obturator insertion.

Soft tissue inflammation (78 %) was the most common MRI features of enthesitis, BME (16 %) was less frequently seen. At two enthesitis sites, BME was seen concomitant with soft tissue inflammation (6 %).

In almost three quarters (74 %) of the patients with pelvic enthesitis, concomitant sacroiliac joint involvement was detected on MRI (Table 4). Conversely, of the 45 patients in our study with sacroiliitis on MRI, 17 (38 %) showed pelvic enthesitis.

**Table 4:** Presence of pelvic enthesitis correlated to the presence of sacroiliitis on MRI

	N	Sacroiliitis + (%)	Sacroiliitis - (%)
enthesitis +	23	17 (74 %)	6 (26 %+)
enthesitis -	120	28 (69 %)	92 (31 %)

(N = number of patients with enthesitis, MRI = Magnetic Resonance Imaging)

The proportion of patients with sacroiliitis was significantly greater in patients with enthesitis than those without (Table 4). This high correlation between enthesitis and sacroiliitis was statistically significant (*p* < 0.001 by Fisher Exact Test).

### Diagnostic value of enthesitis for ERA

Pelvic enthesitis was seen in 23 of 143 (16 %) patients, of whom 12/23 (52 %) patients were diagnosed with ERA and 11/23 (48 %) did not fulfil the ILAR classification. In 2/11 ILAR-negative patients however, ERA was suspected based on the expert opinion of the pediatric rheumatologists. The sensitivity (SN) and specificity (SP) of pelvic enthesitis for diagnosis of ERA were calculated, the presence of more sites of enthesitis on a single MRI study further increases the specificity as illustrated in Table 5.

**Table 5:** Sensitivity and specificity of pelvic enthesitis for the diagnosis of ERA correlated to the number of sites of enthesitis demonstrated on MRI of the SI joints

	N	SN % (CI)	SP % (CI)
enthesitis	23	20 (11.6–33.7)	87 (77.6–93.0)
1 site	16	17 (9.0–29.8)	93 (84.7–97.0)
2 sites	6	3 (0.6–12.9)	95 (87.7–98.5)
3 sites	1	/	99 (92.7–99.9)

(N = number of patients; SN = sensitivity, SP = Specificity, CI = Wilson score 95 % Confidence Interval)

The correlation between pelvic enthesitis and clinically diagnosed ERA was weaker than between enthesitis and sacroiliitis. Patients with pelvic enthesitis on MRI were not significantly more likely to have ERA than others (*p* = 0.25 by Fisher Exact Test).

**Fig. 8:**
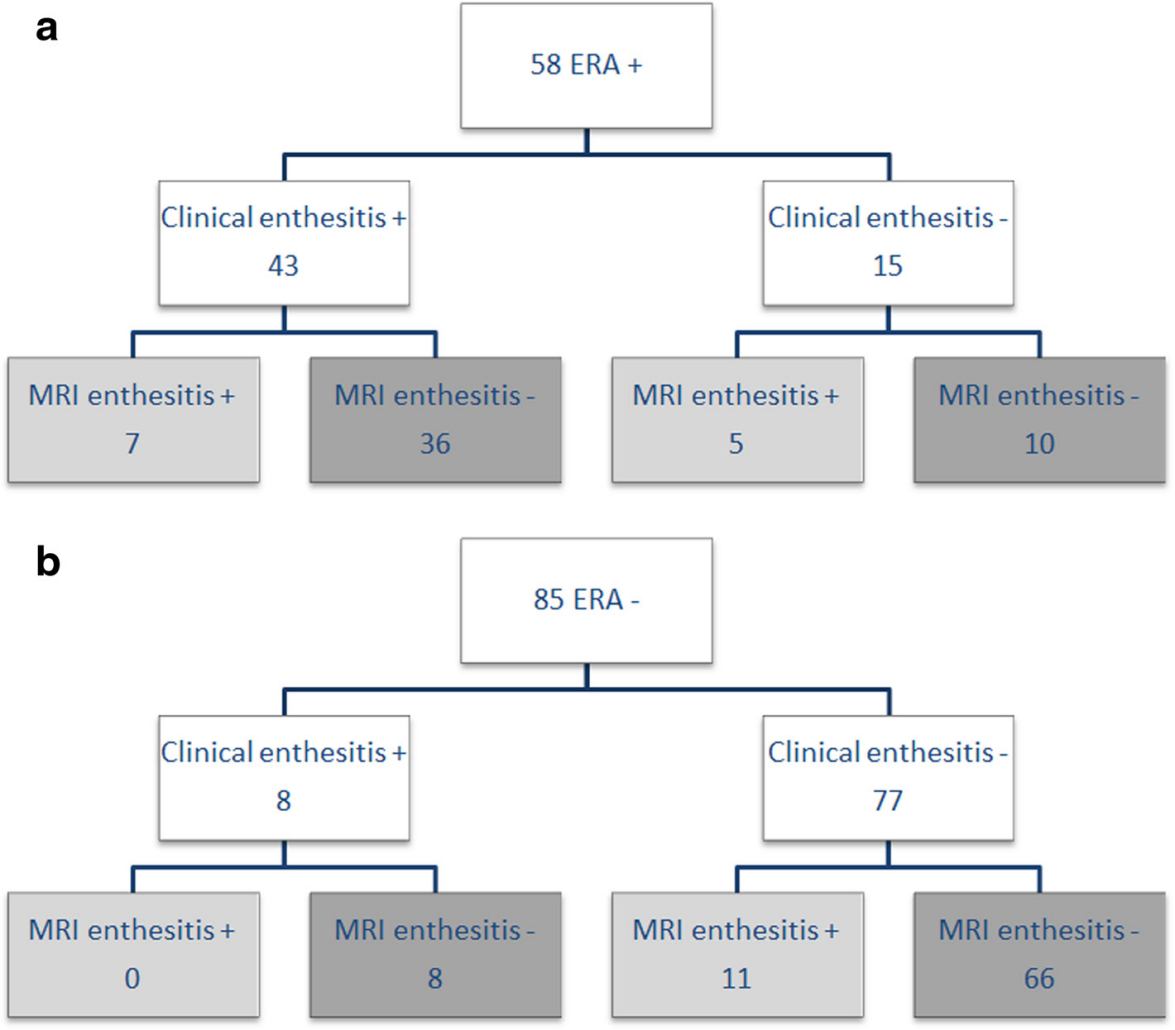
The number of patients with or without clinical enthesitis, correlated to pelvic enthesitis on MRI shown separately for the two groups ERA+ and ERA-. (MRI = Magnetic Resonance Imaging, ERA = Enthesitis Related Arthritis)

In Fig. 8, the number of patients with and without clinical enthesitis, correlated to pelvic enthesitis seen on MRI is shown for the two groups ERA+ and ERA- separately.

In total, of the 51 patients with clinical enthesitis, 7 (14 %) also demonstrate pelvic enthesitis on MRI. Of the 92 patients without clinical enthesitis, pelvic enthesitis was seen on MRI in 16 of 92 (17 %) patients.

In 7 of 11 ERA negative patients without clinical enthesitis but with pelvic enthesitis on MRI, there could have been a change in diagnosis according to the ILAR classification, if pelvic enthesitis on MRI was included in the criteria. One maior criterion (arthritis) was already fulfilled in 2 patients, 5 patients had two or more minor criteria, so if pelvic enthesitis seen on MRI was included, these 7 patients would have fulfilled the ILAR criteria for ERA.

## Discussion

The aim of our study was to determine the prevalence and diagnostic value of pelvic enthesitis on MRI of the SI-joints in ERA. Enthesitis is a primary clinical criterion in ERA, the diagnosis is based on palpable tenderness at insertion sites alone [12]. The deep-seated entheses are difficult to palpate, and some entheses are near a joint, where it may be difficult to differentiate a palpable pain as enthesitis or joint synovitis [19]. Nevertheless, ILAR criteria for ERA use a clinical, not MRI, diagnosis of enthesitis in the definition [7].

In our study, we found pelvic enthesitis on MRI in 21 % of patients with ERA and in 13 % of patients without ERA. The most commonly affected entheses were the entheses around the hip (SP 95 %) and the retroarticular interosseous ligaments (SP 94) which both had a high specificity.

In previous work, Lin et al. also concluded that enthesitis diagnosed by pelvic MRI may be a specific finding in JSpA [20]. Yilmaz et al. demonstrated a high correlation between juvenile SpA and pelvic enthesitis. They also stated that enthesitis could be an unaccompanied early finding of juvenile SpA. Adolescents with early and undiagnosed SpA, lacking a typical clinical history, may present with enthesitis as single finding on MRI of the SI joints [21].

In our series, one girl had retro-articular enthesitis as only MRI finding, developing definite sacroiliitis on follow-up scans. Overall, in our study the presence of enthesitis was highly insensitive for ERA and its presence per se was also nonspecific.

We demonstrated that the concomitant presence of more than 1 site of enthesitis increases the specificity for diagnosis of ERA, to 95–99 % for 2 or more sites. Although uncommon, detection of multiple inflamed entheses therefore suggests a relatively high risk of associated ERA. This corresponds well to findings in adults with SpA, where Jans et al. reported similar findings, although in adults the presence of any pelvic enthesitis had a high specificity for the diagnosis of SpA [22].

The low sensitivity of enthesitis for ERA is not surprising, given that ERA usually presents with peripheral enthesitis or arthritis, and axial involvement may come later in the disease course [4, 5, 7] – our field of view did not include peripheral entheses which may be more clinically obvious, and enthesitis at the visible sites on MRI may have faded long before the axial inflammation causing pain leading to MRI. Another possible explanation for the low association between pelvic enthesitis and ERA is the difficult differentiation between enthesitis and tendinitis. This remains a difficult problem as the signal is very similar. This may be an explanation in some of the enthesitis (or high signal) cases in the non-ERA group, some high signal entheses might be tendinitis cases. According to the ASAS definition, the difference cannot be made. Also, we used the ILAR criteria as golden standard. In our study, some patients were clinically suspected to have ERA according to an expert opinion of our pediatric rheumatologists, but did not fulfil the ILAR criteria, or had exclusion criteria. Some patients may not yet fulfil the ILAR criteria at this moment, but will do later on in life. A single diagnostic or classification system representative for juvenile SpA is still needed. This remains a hot topic in pediatric rheumatology, but this discussion lies beyond the purpose of this paper.

In our study, we found a statistically significant (*p* < 0.001) correlation between pelvic enthesitis and sacroiliitis. Of the 23 patients with pelvic enthesitis, 17 (74 %) also had signs of sacroiliitis. Yilmaz et al. found sacroiliitis in all of the 11 patients with low back pain in his study, and in 8/11 soft tissue edema or inflammation was also seen, leading them to conclude enthesitis could be an important finding [21]. Lin et al. found pelvic enthesitis in 41 % of patients with sacroiliitis [20], very similar to the 38 % of patients in our study. Pagnini et al. showed that in patients with ERA, the number of affected entheses and joints at onset can predict sacroiliitis at follow-up [15].

In our study, there was no good correlation of the pelvic enthesitis MR findings with the clinical finding of enthesitis. However in our study, all enthesitis diagnosed clinically was peripherally. In 16 of 92 (17 %) patients without clinical enthesitis, pelvic enthesitis was detected on MRI. As beforementioned, deep-seated entheses such as the pelvic entheses are difficult to address clinically, and MRI could be of value. In our study, 7 patients could have had a change in diagnosis according to the ILAR classification, if enthesitis on MRI was included in the criteria. However the diagnosis will not change at the moment since imaging is not included in the diagnostic criteria, which is a difficulty in studying this features.

In our study, we used semicoronal T1 and semicoronal and axial fluid-sensitive MR images of the SI joints for the evaluation of the deep pelvic entheses, as is recommended by the ASAS handbook guidelines [13]. Other MRI studies in patients with SpA or ERA also used axial images in combination with semicoronal imaging for evaluation of the sacroiliac joints, and thus the entheses [10, 15, 21–23]. Rachlis et al. evaluated findings on whole-body MRI (WB-MRI) in patients with ERA compared to clinical examination, and found that MRI was superior to clinical exam for the hips, SI-joints and spine. For enthesitis, clinical examination overestimated disease activity in the periphery, making WB-MRI an important tool to evaluate entheseal disease [24].

Several studies investigated the evaluation of entheses with power Doppler Ultrasonography (US) [25, 26], all concluding US can be useful for the assessment of enthesitis. US has the advantages of being cheaper, more easily accessible, mobile, and not requiring sedation in younger children. On the other hand, MRI can depict soft tissue inflammation and joint effusion/bursitis, as well as bone marrow edema, and gives a more complete picture [10]. We did not assess enthesitis by ultrasound in this study.

Compared to other studies, there was a high proportion of girls in our studygroup. Recent classifications on spondyloarthritis, such as the ILAR classification, tended to focus less on the presence of HLA B27 but added more clinical aspects of peripheral arthritis as well as extra–articular manifestations. More females fitted into these more recent classifications, resulting in an increasing proportion of HLA-B27 negative girls in SpA and ERA. Our study shows the same trend, but this should be carefully addressed, since our series was initially not based on any classification, but in second hand checked for the fulfilment of criteria. The sex ratio in this series therefore is pure coincidental and should not lead to any conclusion.

There are some limitations to our study. A major limitation is the retrospective nature of the study, resulting in a lack of clinical data. For many patients our pediatric rheumatologists could not determine a specific diagnosis based on the clinical files. Also, the pelvic entheses were not assessed separately on clinical exam. Another important limitation is the relative small number of patients. We did not have a control group of asymptomatic children. However, this is an inherent bias in retrospective studies. MRI was also the only imaging technique, without correlation with radiography or ultrasound. There was no contrast administration. Furthermore, only active enthesitis was recorded and not the structural, non-inflammatory changes of healed enthesitis. The patient population represented referrals from a single tertiary centre; referral patterns for sacroiliitis may vary elsewhere. Additional data from larger, prospective studies are necessary with good, organised clinical exam and a control population, to confirm our findings regarding the prevalence and diagnostic value of pelvic enthesitis on MRI of the SI joints in ERA.

## Conclusions

Our study presents the features of pelvic enthesitis on MRI of the SI joints in children with ERA. Pelvic enthesitis may be present in children with or without clinically evident peripheral enthesitis. There is a high correlation between pelvic enthesitis and sacroiliitis on MRI of the sacroiliac joints in children. As pelvic enthesitis indicates active inflammation, it may play a role in assessment of the inflammatory status, and for timing and aggressiveness of therapy. Therefore, it should be carefully sought and noted by radiologists examining MRI of the sacroiliac joints in children.
